# Synbiotic Effect of *Bifidobacterium Lactis* CNCM I-3446 and Bovine Milk-Derived Oligosaccharides on Infant Gut Microbiota

**DOI:** 10.3390/nu12082268

**Published:** 2020-07-29

**Authors:** Benoît Marsaux, Pieter Van den Abbeele, Jonas Ghyselinck, Guénolée Prioult, Massimo Marzorati, Biljana Bogićević

**Affiliations:** 1 Center for Microbial Ecology and Technology (CMET), Department of Biotechnology, Faculty of Bioscience Engineering, Ghent University, Coupure Links 653, 9000 Ghent, Belgium; benoit.marsaux@prodigest.eu (B.M.); massimo.marzorati@prodigest.eu (M.M.); 2 ProDigest BV, Technologiepark 82, 9052 Ghent, Belgium; Pieter.VandenAbbeele@prodigest.eu (P.V.d.A.); jonas.ghyselinck@prodigest.eu (J.G.); 3 Nestlé Research and Development Konolfingen, Nestléstrasse 3, 3510 Konolfingen, Switzerland; guenolee.prioult@rdko.nestle.com; 4 Nestlé Research, Route du Jorat 57, 1000 Lausanne, Switzerland

**Keywords:** infant gut microbiota, *Bifidobacterium animalis* ssp. *lactis*, BMOS, in vitro fermentation, probiotic, prebiotic, dysbiosis, preculturing, synbiotic, oligosaccharides

## Abstract

Background: This study evaluated the impact of *Bifidobacterium animalis* ssp. *lactis* CNCM I-3446, Bovine Milk-derived OligoSaccharides (BMOS) and their combination on infant gut microbiota in vitro. In addition, a novel strategy consisting of preculturing *B. lactis* with BMOS to further enhance their potential synbiotic effects was assessed. Method: Short-term fecal batch fermentations (48 h) were used to assess the microbial composition and activity modulated by BMOS alone, *B. lactis* grown on BMOS or dextrose alone, or their combinations on different three-month-old infant microbiota. Results: BMOS alone significantly induced acetate and lactate production (leading to pH decrease) and stimulated bifidobacterial growth in 10 donors. A further in-depth study on two different donors proved *B. lactis* ability to colonize the infant microbiota, regardless of the competitiveness of the environment. BMOS further enhanced this engraftment, suggesting a strong synbiotic effect. This was also observed at the microbiota activity level, especially in a donor containing low initial levels of bifidobacteria. In this donor, preculturing *B. lactis* with BMOS strengthened further the early modulation of microbiota activity observed after 6 h. Conclusion: This study demonstrated the strong synbiotic effect of BMOS and *B. lactis* on the infant gut microbiota, and suggests a strategy to improve its effectiveness in an otherwise low-*Bifidobacterium* microbiota.

## 1. Introduction

Depending on the mode of delivery, the infant gut is rapidly colonized by microorganisms either from the environment and/or the mother’s vaginal, fecal and skin microbiota [1,2]. Several other factors including prematurity, infant diet (breast milk or formula), hygiene and use of antibiotics will ultimately affect the composition of the infant gut microbiota [3,4,5,6]. Epidemiological studies have identified associations between antibiotic usage in early infancy and the occurrence of diseases such as obesity, diabetes and asthma later in life [7,8]. Thus, a large and growing number of studies implicate a potential role for microbiota imbalance (dysbiosis) in numerous diseases [9,10,11].

In order to support the normal development of the gut microbiota of infants susceptible to dysbiosis, the use of probiotics and/or prebiotics in infant formula has gained a lot of attention [12]. While probiotics are live bacteria that confer a health benefit (FAO/WHO, 2001; revised by [13]), prebiotics are substrates that selectively stimulate beneficial members of the indigenous gut microbiota ([14]; revised by [15]). Specifically, Galacto-OligoSaccharides (GOS) are among the most studied [16,17,18] and used prebiotics in or with infant formula. This followed the initial observation by Tanaka and colleagues [19] who reported that GOS stimulated the growth of resident *Bifidobacterium* species in humans. It can ultimately lead to health benefits, as the high abundance of bifidobacteria in infant feces was correlated with reduced risk of infections and allergies [20,21,22,23]. Yet, it has also been suggested that a general bifido-stimulating effect could be insufficient, and that species-specific stimulation is needed to reach the desired health benefit [24]. Therefore, there is a growing interest in the development of synbiotic treatments, where a specific prebiotic selectively increases the growth of a coadministered probiotic strain with well-characterized health benefits [25].

Strains of *Bifidobacterium animalis* ssp. *lactis*, for instance, are widely used probiotics consumed for their beneficial effects through interactions with the host and with other components of the intestinal microbiota [26]. Sazawal and colleagues [27] reported a reduction in bloody diarrhea, days with fever, and the prevalence of ear infection in infants consuming *B. lactis* (HN019) together with GOS. More recently, Bovine Milk-derived OligoSaccharides (BMOS) were proposed as a valid alternative to GOS [28,29,30,31]. BMOS, as described by Meli and colleagues [32], are derived from demineralized whey permeate. It contains GOS and sialylated oligosaccharides (3′- and 6′-sialyllactose) naturally present in cow’s milk. Noteworthy, 3′- and 6′-sialyllactose are similar to Human Milk OligoSaccharides [33] which were proven to be beneficial for infant health [34]. In two clinical trials, the supplementation of infant formulas with *B. lactis* (CNCM I-3446) and BMOS induced a shift towards bifidobacteria-dominated stools, resembling those of breastfed infants [35,36]. The addition of the two ingredients seems therefore to play a key role in the gut microbiota composition. However, it remains unclear whether *B. lactis* and BMOS act individually or in a synbiotic way.

*B. lactis* is a non-host-specific subspecies transmitted between various animals, of which the natural habitat is the gastrointestinal tract [37,38]. Yet, it is often considered as a nonresident species of the human gut [39]. It is indeed only sparsely detected in infant feces [40,41]. As a matter of fact, *B. lactis* is supposedly unable to use the carbon resources present in the human gut, as it has a limited number of hypothetical glycosyl hydrolases and carbohydrate pathways [42,43,44]. However, some *B. lactis* strains have been shown to be able to metabolize GOS structures [45]. Therefore, in order to ensure its engraftment in infant gut microbiota, we propose first to look at the response of *B. lactis* strain CNCM I-3446 to BMOS. We then develop hereafter a strategy that consists of preculturing the bacteria with BMOS prior to coadministration with BMOS. By doing so, we hopefully prime *B. lactis* to efficiently metabolize BMOS, and provide *B. lactis* with a competitive advantage over other microbes to survive in the presence of BMOS in very competitive environments.

While clinical trials are essential to demonstrate health effects in a particular group of people, in vitro studies are useful to provide detailed insights into mechanisms of action since they do not only focus on end-point measurements (through feces analysis). They also allow several formulations to be tested simultaneously in very controlled conditions. Therefore, in this study, we used a highly standardized in vitro colonic fermentation model [46] combined with metabolic activity and high-resolution microbial composition analysis (16S rRNA gene-targeted Illumina sequencing) to evaluate the potential efficacy of a novel strategy to improve infant gut health, while taking into account potential infant interindividual variability. This strategy includes combining BMOS with the well-characterized *Bifidobacterium animalis* ssp. *lactis* strain CNCM I-3446 [47,48], which already proved to be beneficial in infant gut [36]. In order to discriminate the prebiotic effects of BMOS from the probiotic effects of *B. lactis* and synbiotic effects of their combination, all three conditions were tested separately. Finally, we assessed whether the synbiotic effect of *B. lactis* and BMOS could be enhanced by priming *B. lactis* with BMOS prior to coadministration.

## 2. Materials and Methods

### 2.1. Chemicals and Carbohydrates

All chemicals were obtained from Merck (Darmstadt, Germany) unless stated otherwise. Nestlé Research and Development Konolfingen (Konolfingen, Switzerland) provided Bovine Milk-derived OligoSaccharides (BMOS), a carbohydrate mixture generated from demineralized whey permeate which contains Galacto-OligoSaccharides (GOS) and other naturally present oligosaccharides, such as 3′- and 6′-sialyllactose. The manufacturing process of BMOS is described by Meli et al. [32].

### 2.2. Bacterial Strains and Growth

*Bifidobacterium animalis* ssp. *lactis* strain CNCM I-3446 (*B. lactis*; Bl) came from the Nestlé Culture Collection (Lausanne, Switzerland) but originated from the DSM (German culture collection; Braunschweig, Germany). It was grown in media made of either dextrose (Pre-Dextrose Bl) or BMOS (Pre-BMOS Bl) at 2.8%, yeast-derived amino acids at 3% and vitamin C. Media were inoculated with 10^7^ colony-forming unit per mL (cfu/mL) and incubated at 37 °C in a water bath. The iCinac pH monitoring system (AMS Alliance, Rome, Italy) was used for monitoring acidification during fermentation, which can be used as an indication for the growth of lactic acid-producing bacteria [49]. After fermentation, acidification curves were compared to ascertain their similarity. Finally, cell counts were determined by flow cytometry as described in ISO 19344:2015 (IDF 232) by the PI/Syto24 method. Consequently, a volume containing 1 × 10^9^ active fluorescent unit (afu) live bacteria was twice washed with Phosphate-Buffered Saline (PBS) prior to being frozen at −80 °C with 20% glycerol.

### 2.3. Experimental Approach

In a first experiment, the bifidogenic response to BMOS of 10 three-month-old infant fecal microbiota was assessed in an in vitro fecal batch incubation system (described below). This prescreening experiment consisted of a short-term colonic incubation in which BMOS was added to the sugar-depleted nutritional medium, and compared to a control incubation (Control). The pH, gas, Short-Chain Fatty Acid (SCFA), lactate production and targeted-quantitative Polymerase Chain Reaction (qPCR) for *Bifidobacterium* quantification were evaluated to select the two most appropriate donors for the main experiment. The latter consisted of testing five formulations against the Control: (i) prebiotic alone (BMOS), (ii–iii) probiotic alone (Pre-Dextrose Bl or Pre-BMOS Bl) or (iv–v) synbiotic (Pre-Dextrose Bl + BMOS or Pre-BMOS Bl + BMOS), for which the pH, gas, SCFA, lactate, ammonium production and 16S rRNA gene sequencing were used to assess the overall microbial metabolic activity and composition change.

### 2.4. Description of the In Vitro Fecal Batch Incubations of 10 Three-Month-Old Infants

Fecal batch fermentations were performed for different test conditions against 10 three-month-old infant gut microbiota (donors 1 to 10) according to Van den Abbeele and colleagues [46]. Briefly, colonic background medium (yielding a final concentration of K_2_HPO_4_ 4.7 g/L; KH_2_PO_4_ 14.7 g/L; NaHCO_3_ 1.8 g/L; yeast extract 1.8 g/L; peptone 1.8 g/L; mucin 0.9 g/L; cysteine 0.5 g/L; polyoxyethylene (20) sorbitan monooleate 1.8 mL/L in the reactors) was added to reactors, already containing *Bifidobacterium animalis* ssp. *lactis* strain CNCM I-3446 (dose corresponding with 1.5 × 10^7^ CFU/mL at the start of incubation) and BMOS (dose corresponding with 5 g/L at the start of incubation) when applicable. BMOS dose was chosen for its physiological relevance, based on the usual dosage of GOS in infant formula products leading to health benefits [50]. The bottles were sealed with rubber stoppers and anaerobiosis was obtained by flushing with N_2_. Subsequently, 1 mL of a healthy infant fecal inoculum was added to a total volume of 63 mL incubation fluid (composed as described above), prepared by making a 7.5% (w/v) suspension of a freshly collected fecal sample in anaerobic phosphate buffer (K_2_HPO_4_ 8.8 g/L; KH_2_PO_4_ 6.8 g/L; sodium thioglycolate 0.1 g/L; sodium dithionite 0.015 g/L). At that point, the actual incubation started for a period of 48 h during which temperature was controlled at 37 °C and homogeneity was ensured by a shaker (90 rpm). All experiments were performed in either simplicate (prescreening experiment) or triplicate (main experiment).

### 2.5. Microbial Metabolic Activity: pH, Gas Production, Short-Chain Fatty Acids (SCFA) and Ammonium

Simplicate measurement was performed for pH (Senseline F410; ProSense, Oosterhout, The Netherlands), gas pressure (Hand-held pressure indicator CPH6200; Wika, Echt, The Netherlands), lactate and SCFA. Samples were taken at the start of the incubation and after 48 h for the prescreening experiment, while also after 6 h for the main experiment only. Ammonium production was assessed at the beginning and at 48 h for the main experiment only. SCFA levels, including acetate, propionate, butyrate and branched SCFAs (isobutyrate, isovalerate and isocaproate) were measured as described by De Weirdt and colleagues [51], although no branched SCFAs were quantifiable in this study. Lactate quantification was performed using a commercially available enzymatic assay kit (R-Biopharm, Darmstadt, Germany) according to the manufacturer’s instructions. Ammonium analysis was performed as described by Van de Wiele and colleagues [52]. Briefly, the ammonium in the liquid samples was quantified by initially performing a steam distillation. Subsequently, the ammonium in the distillate was measured by titration with HCl.

### 2.6. Microbial Community Analysis by qPCR

Samples from the fecal slurry of the 10 donors used for the prescreening experiment, as well as samples after 48 h of incubation from the batch fermentation in the Control and BMOS conditions were collected for evaluating the total amount of bifidobacteria by qPCR. Briefly, DNA was isolated using the protocol as described by Vilchez-Vargas and colleagues [53], starting from cell pellets from 1 mL sample aliquots. The bifidobacteria 16S rRNA gene copy number was determined by qPCR as described by Rinttilä and colleagues [54], with the Bif243F (5′-TCGCGTCYGGTGTGAAAG-3′) and the Bif243R (5′CCACATCCAGCRTCCAC-3′) using a QuantStudio 5 Real-Time PCR system (Applied Biosystems, Foster City, CA, USA). Each sample was analyzed in simplicate. Results are reported as log (16S rRNA gene copies/mL).

### 2.7. Microbial Community Analysis by 16S rRNA Gene Sequencing

Samples were collected after 48 h of incubation from the batch fermentation of the main experiment in the Control, BMOS, *B. lactis* and synbiotic conditions for Donors 1 and 8 for in-depth microbial community analysis. DNA was extracted as described before and samples were sent out to LGC Genomics (Teddington, Middlesex, UK) for next-generation 16S rRNA gene amplicon sequencing of the V3–V4 region. The 341F (5′-CCTACGGGNGGCWGCAG-3′) and 785R (5′-GACTACHVGGGTATCTAAKCC-3′) primers were used according to De Paepe and colleagues [55], with the reverse primer being adapted from Klindworth and colleagues [56] to increase coverage. Quality control PCR was conducted using Taq DNA Polymerase with the Fermentas PCR Kit according to the manufacturers’ instructions (Thermo Fisher Scientific, Waltham, MA, USA). The DNA quality was verified by electrophoresis on a 2% (*w/v*) agarose gel for 30 min at 100 V.

### 2.8. Bioinformatics Analysis of Amplicon Data

The mothur software package (v. 1.39.5) and guidelines were used to process the amplicon data generated by LGC Genomics as previously described in De Paepe et al. (2017) [57]. An Operational Taxonomic Unit (OTU) is hereinafter defined as a collection of sequences with a length between 402 and 427 nucleotides that are found to be more than 97% similar to one another in the V3–V4 region of their 16S rRNA gene after applying Opticlust clustering [57,58,59,60,61]. Taxonomy was assigned using the RDP version 16 and silva.nr_v123 database [62,63,64]. The shared file, containing the number of reads observed for each OTU in each sample, was loaded into Microsoft^®^ Excel^®^ 2016 MSO (16.0.11901.20070, Redmond, WA, USA). Reads occurring only 5 times in all samples were removed, as they were supposedly artifacts or bacteria that were not having any biological impact. For the most abundant OTUs, the sequences retrieved from a 3% dissimilarity level fasta file obtained in mothur were classified through the RDP web interface using the RDP SeqMatch tool. The database search was restricted to type strains with only near-full-length and good quality sequences. The sequences were blasted in NCBI against the 16S rRNA gene sequences, selecting only type material, with optimization of the BLAST algorithm for highly similar sequences (accession date: December 2018) [62,64,65]. Although identification to the species level based on short 300 bp reads may involve some ambiguity, the most likely species classification of a few interesting OTUs is reported in the results sections. In the event of inconsistencies in the results of the RDP SeqMatch tool and NCBI BLAST, no species-level classification is provided. The results are presented as proportional values.

### 2.9. Statistics

All statistical analyses were performed in GraphPad Prism version 8.2.0 (435) for Windows (GraphPad Software, San Diego, CA, USA). All formal hypothesis tests were conducted on the 5% significance level (α = 0.05).

A comparison of the data of the Control and BMOS conditions on microbial metabolic and composition markers of the 10 donors was done by calculating the average per condition and then by performing a paired sample t-test. The normal distribution of the data and residuals was checked based on visual inspection of QQ-plots. In case the assumption of normality was not reached, a Wilcoxon matched-pairs signed-rank test was performed instead.

Comparison of the data of the Control, BMOS, prebiotic and synbiotic conditions on microbial metabolic and composition markers of Donors 1 and 8 was done by performing a one-way ANOVA with Tukey’s Honestly Significant Difference (HSD) post hoc test. The approximation of the normal distribution of the data was assumed due to the small sample size [66], and the homoscedasticity across the samples was checked by plotting the residuals, although the sample size was equal.

### 2.10. Ethics

Legal representatives of all subjects gave their informed consent for inclusion before the study was initiated. The study was conducted in accordance with the Declaration of Helsinki, and fecal samples were collected according to the ethical approval of the University Hospital Ghent (reference number B670201836585).

## 3. Results

### 3.1. BMOS Significantly Modulated Gut Microbial Activity and Composition from 10 Infants

**Figure 1:**
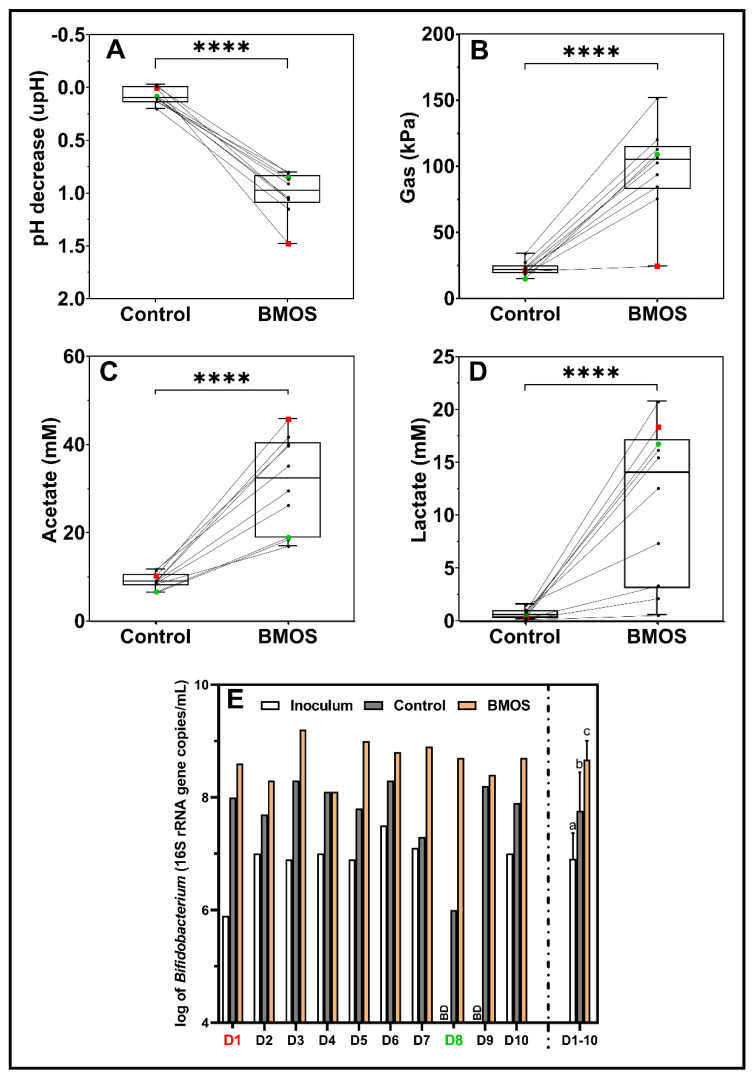
Microbial metabolic activity in terms of pH decrease (upH) (**A**), gas production (kPa) (**B**), acetate production (mM) (**C**) and lactate production (mM) (**D**), as well as the absolute abundance of bifidobacteria (log (16S rRNA gene copies/mL)) (**E**) upon a 48 h fermentation of 5 g/L Bovine Milk-derived OligoSaccharides (BMOS, *n* = 1) by an infant fecal microbiota of 10 different donors (D1–D10), versus their respective blank controls (Control, *n* = 1). (**A**–**E**) Donors 1 and 8 are colored in red and green respectively. (**E**) The average and standard deviation of the 10 donors (D1–10) are shown for *Bifidobacterium* levels. Significant differences between the average Control and BMOS, as tested with a paired sample t-test, are indicated with different letters (a, b, c; *p* < 0.05) or asterisks (****, *p* < 10^−4^). BD: Below Detection (i.e., <1 × 10^5^).

BMOS alone consistently significantly decreased pH and increased gas production (markers for intensity of microbial activity) versus control incubations (Control, *p* < 0.0001; Figure 1A,B). pH decreased by 1.00 upH on average in the presence of BMOS after 48 h of incubation (versus 0.08 upH for Control). Gas pressure increased on average from 22.6 kPa (Control) to 98.4 kPa in response to BMOS.

**Figure A1:**
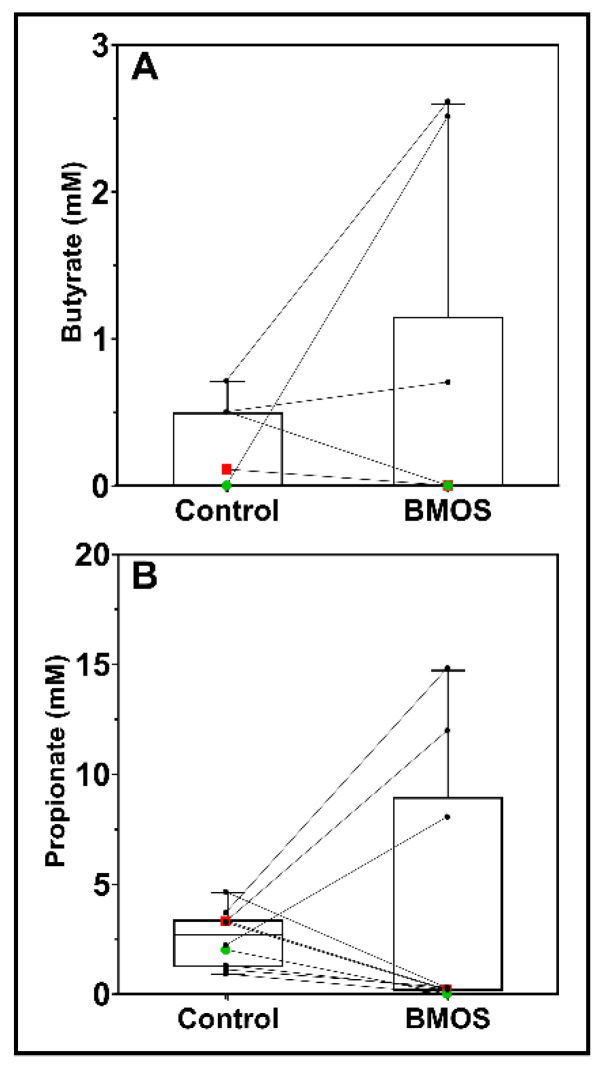
Microbial metabolic activity in terms of butyrate production (mM) (**A**) and propionate production (mM) (**B**) upon a 48 h fermentation of 5 g/L BMOS (*n* = 1) by infant fecal microbiota of 10 different donors, versus their respective blank controls (Control, *n* = 1). Donors 1 and 8 are colored in red and green, respectively. No significant differences between the average Control and BMOS were obtained, as tested with a paired sample t-test (*p* > 0.05).

In addition, while acetate and lactate levels were similar among all control incubations (i.e., 9.2 mM and 0.7 mM on average, respectively.), BMOS consistently and significantly (*p* < 0.0001) increased levels of both metabolites (31.3 mM and 11.4 mM on average, respectively.) with the intensity of the increases being donor-dependent (Figure 1A,B). Branched SCFAs (isobutyrate, isovalerate and isocaproate) were not detected (data not shown) as expected in preweaning infants [67], while effects on propionate and butyrate production were minor and donor-dependent (Figure A1A,B).

Bifidobacteria were quantified by qPCR prior to incubation (i.e., inoculum) and after 48 h of incubations for both BMOS treatment and Control. BMOS consistently significantly increased the absolute amount of bifidobacteria over the 10 donors tested, reaching on average 8.7 log (16S rRNA gene copies/mL) versus 7.9 log in Control (*p* < 0.01) with the extent of the bifidogenic effect being donor-dependent (Figure 1E). While levels of bifidobacteria were below the detection limit in Donors 8 and 9 (i.e., <5 log (16S rRNA gene copies/mL)), it reached levels similar to the average after BMOS treatment, whereas it remained low in the Control for Donor 8.

Although the effect of BMOS on microbiome composition and microbiota activity seemed overall consistent across the 10 donors, some donors responded more strongly than others. Donor 1 (in red) responded strongly to BMOS as observed by high acetate and lactate productions leading to a high pH decrease (Figure 1). On the contrary, Donor 8 (in green), containing low levels of bifidobacteria (Figure 1E), happened to produce mild levels of acetate and lactate triggering the mildest pH decrease in the presence of BMOS (Figure 1). Hence, among the 10 tested donors, Donors 1 and 8 were selected for the in-depth investigation of the potential synbiotic effect between *B. lactis* and BMOS.

### 3.2. BMOS Significantly Increased B. lactis Engraftment as Tested for Two Infant Microbiomes

**Figure 2:**
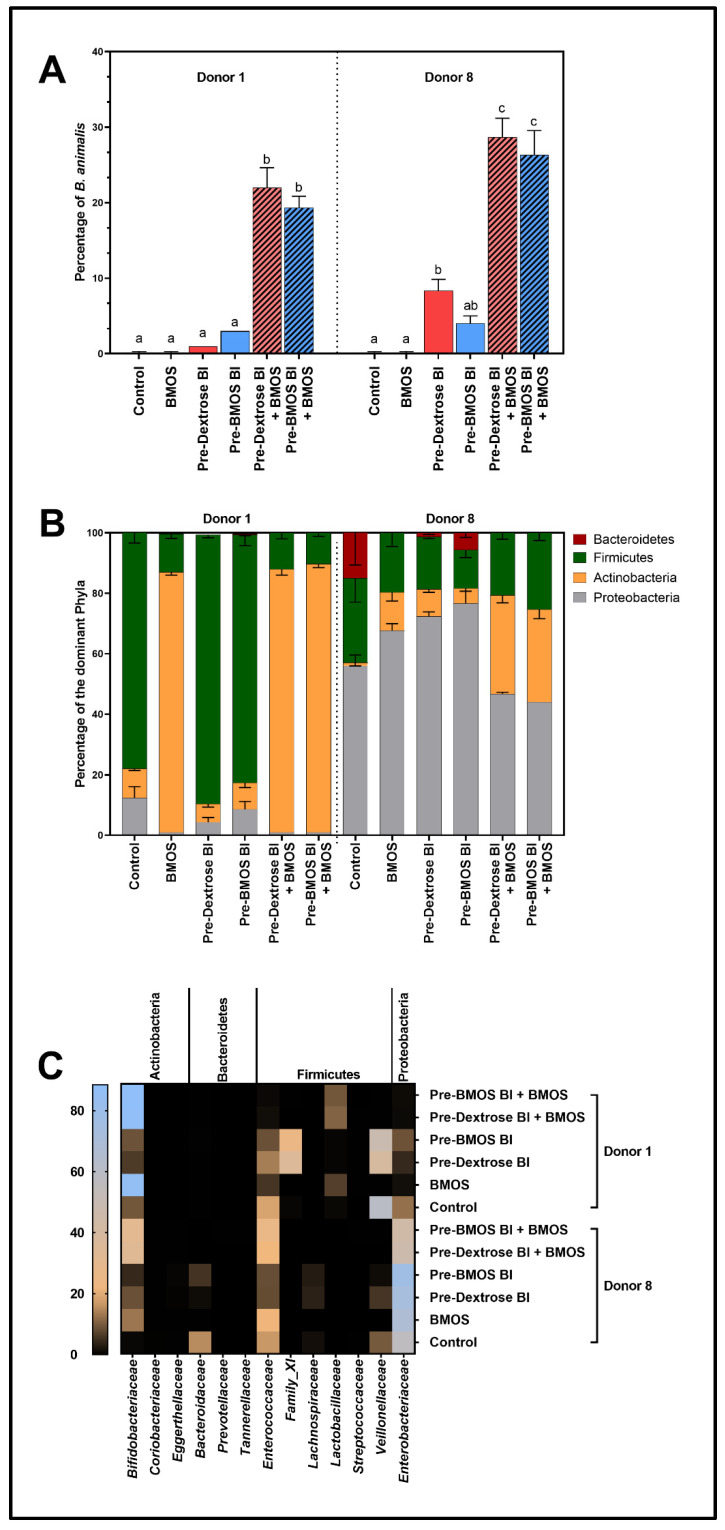
Mean (± standard deviation) relative abundance (%) of the dominant phyla (**B**) (through a heat map), families (**C**) and specifically of *B. animalis* (**A**) upon a 48 h fermentation of 5 g/L BMOS containing 1.5 × 10^7^ CFU/mL of *B. lactis* previously grown on either Dextrose (Pre-Dextrose Bl + BMOS, *n* = 3) or BMOS (Pre-BMOS Bl + BMOS, *n* = 3), by an infant fecal microbiota of Donors 1 and 8, versus their respective controls (Control, Pre-Dextrose Bl and Pre-BMOS Bl, *n* = 3). Significant differences between the different formulations within each donor are indicated with different letters (a, b, c), as tested with a one-way ANOVA with Tukey’s HSD post hoc test (*p* < 0.05). In contrast, when at least one letter is shared between two treatments, there was no significant difference between these groups.

16S rRNA-targeted sequencing analysis provided insight into the impact of five formulations on the microbial compositions of two selected donors—Donors 1 and 8—at the end of the short-term colonic incubations (48 h). The formulations included (i) BMOS (= prebiotic), (ii–iii) *B. lactis* (Bl) pregrown with BMOS (Pre-BMOS Bl) or Dextrose (Pre-Dextrose Bl = probiotic) and (iv–v) pregrown *B. lactis* (Pre-BMOS Bl and Pre-Dextrose Bl) together with BMOS (= synbiotic). For both infant donors tested, one specific operational taxonomic unit (OTU) related to *B. animalis* was exclusively and constantly detected upon dosing with formulations containing *B. lactis* (Figure 2A). Hence, although 16S rRNA-target sequencing analysis only provides genus-level identification [68], this OTU likely corresponded to the probiotic contained in the formulations. *B. lactis* successfully engrafted the microbiota in both donors (i.e., between 1% and 8%). Interestingly, the relative abundance of *B. lactis* was higher (i.e., between 19% and 29%) when combined with BMOS, suggesting a potential strong synbiotic effect of BMOS on *B. lactis* engraftment. Further, the relative abundance of *B. lactis* was found more pronounced in Donor 8 than in Donor 1, which might imply better colonization of *B. lactis* in microbiota containing low levels of bifidobacteria at the start (Figure 1E and Figure 2C). However, preconditioning of *B. lactis* with BMOS (Pre-BMOS Bl) did not lead to improved engraftment regardless the presence of BMOS (with or without) and the donor (Donors 1 or 8; Figure 2A). Further quantitative analysis would be required to confirm those findings.

### 3.3. Microbiota Composition Differently Modulated by BMOS, B. lactis and Synbiotics

At phylum level, both BMOS and the two synbiotics strongly increased Actinobacteria (containing *Bifidobacteriaceae*) within the microbiota of Donor 1 (from ~10% to 86%), and this exclusively at the expense of Firmicutes relative abundance (decreased from ~78% to 13%; Figure 2B). For Donor 8, the main phylum in the Control, i.e., Proteobacteria (containing opportunistic pathogens) was stimulated by BMOS and *B. lactis* given alone (from ~56% to 67–77%), but not by the synbiotics (reaching ~44–46%). In contrast, due to strong stimulation of Actinobacteria (from ~1% to 31–33%), the synbiotic formulations decreased not only Bacteroidetes (from ~12% to 0%) but also Proteobacteria (to ~45%) relative abundance.

The composition was further refined at family, genus and OTU level, stressing that microbial composition was strongly altered by all formulations (Figure 2C and Table 1 and Table 2). For Donor 1, both BMOS and the synbiotic treatments increased *Bifidobacteriaceae* levels, related to stimulation of specific OTUs (Table 1). The synbiotics and especially BMOS drastically increased an OTU related to *B. breve* (e.g., from ~3% to 74% with BMOS), whereas only the synbiotics (and to a lesser extent the probiotics) significantly increased an OTU related to *B. animalis* (e.g., from 0% to ~22% with Pre-Dextrose Bl and BMOS). Finally, an OTU related to *B. bifidum* slightly increased with BMOS but decreased upon probiotic administration. Further, the relative abundance of all families belonging to the Firmicutes decreased upon treatment with synbiotics, with the exception of *Lactobacillaceae* (OTU related to *L. fermentum*). In addition, an OTU related to *V. parvula/dispar* was enriched in the presence of BMOS-grown *B. lactis* (Pre-BMOS Bl), but not by Dextrose-grown *B. lactis* (Pre-Dextrose Bl).

Similarly to Donor 1, overall *Bifidobacteriaceae* levels were increased by the treatments in Donor 8, with OTU-specific variations (Table 2). The probiotics given alone increased an OTU related to *B. animalis* (e.g., from 0% to ~8% with Pre-Dextrose Bl), while BMOS alone increased OTUs related to both *B. longum* and *B. adolescentis*. The synbiotics increased OTUs related to *B. longum* but most strongly the one related to *B. animalis* (e.g., from 0% to ~29% with Pre-Dextrose Bl + BMOS). Further, BMOS or the probiotics alone specifically increased an OTU related to *E. coli*. *Clostridiaceae* slightly increased with the probiotics while decreased with BMOS and the synbiotics. The opposite was observed for the *Enterococcaceae* (OTU related to *Enterococcus faecalis*). Finally, next to an OTU related to *Klebsiella oxytoca/michiganensis*, the following families’ relative abundance consistently decreased with the various treatments: *Bacteroidaceae* (OTU related to *B. ovatus*), *Veillonellaceae* (OTU related to *V. dispar*) and *Coriobacteriaceae* (OTU related to *Collinsella aerofaciens*).

Altogether, our data showed that microbiota composition was overall differently modulated by BMOS, *B. lactis* and synbiotics in both Donors 1 and 8. However, major changes only occurred in the presence of synbiotics. Growing *B. lactis* on BMOS (Pre-BMOS Bl) or Dextrose (Pre-Dextrose Bl) did not impact the effect of *B. lactis* given alone or as synbiotics (Table 1 and Table 2).

### 3.4. Synbiotic Effect on Microbial Community Activity Mainly Seen in Disturbed Microbiome

**Figure 3:**
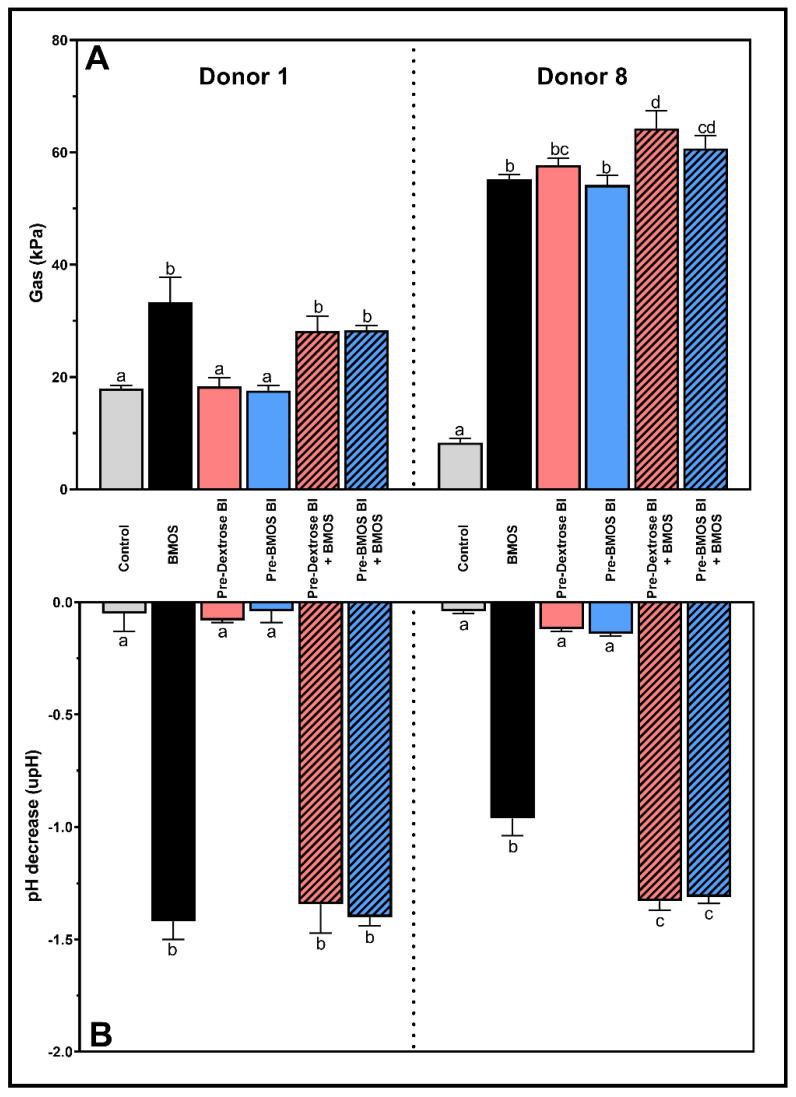
Mean (± standard deviation) microbial metabolic activity in terms of gas production (kPa) (**A**) and pH decrease (upH) (**B**) upon a 48 h fermentation of 5 g/L BMOS containing 1.5 × 10^7^ CFU/mL of *B. lactis* previously grown on either Dextrose (Pre-Dextrose Bl + BMOS, *n* = 3) or BMOS (Pre-BMOS Bl + BMOS, *n* = 3), by the infant fecal microbiota of Donors 1 and 8, versus their respective controls (Control, Pre-Dextrose Bl and Pre-BMOS Bl, *n* = 3). Significant differences between the different formulations within each donor are indicated with different letters (a, b, c, d), as tested with a one-way ANOVA with Tukey’s HSD post hoc test (*p* < 0.05). In contrast, when at least one letter is shared between two treatments, there was no significant difference between these groups.

BMOS alone and the synbiotic formulations significantly decreased pH and increased gas production versus the Control for both donors after 48 h of incubation (*p* < 0.0001; Figure 3A,B). In contrast, probiotic formulations had no impact on pH, nor on gas production for Donor 1, while they increased gas production for Donor 8, reaching similar levels as the other treatments.

**Figure 4:**
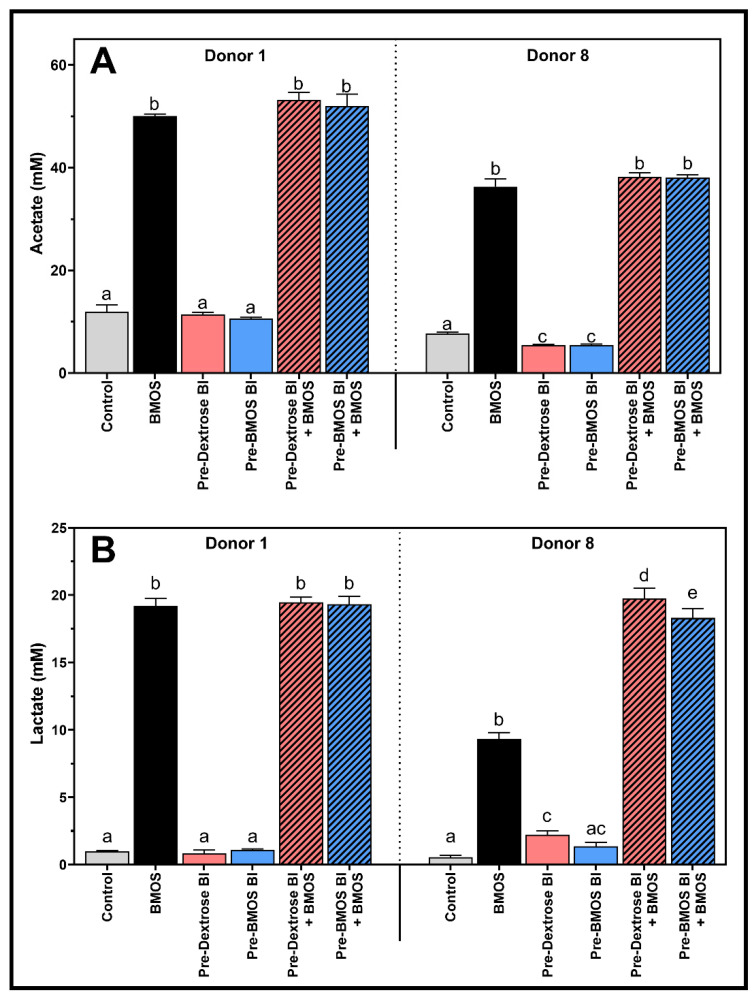
Mean (± standard deviation) microbial metabolic activity in terms of acetate production (mM) (**A**) and lactate production (mM) (**B**) upon a 48 h fermentation of 5 g/L BMOS containing 1.5 × 10^7^ CFU/mL of *B. lactis* previously grown on either Dextrose (Pre-Dextrose Bl + BMOS, *n* = 3) or BMOS (Pre-BMOS Bl + BMOS, *n* = 3), by the infant fecal microbiota of Donors 1 and 8, versus their respective controls (Control, Pre-Dextrose Bl and Pre-BMOS Bl, *n* = 3). Significant differences between the different formulations within each donor are indicated with different letters (a, b, c, d, e), as tested with a one-way ANOVA with Tukey’s HSD post hoc test (*p* < 0.05). In contrast, when at least one letter is shared between two treatments, there was no significant difference between these groups.

BMOS significantly increased acetate and lactate productions versus the Control (*p* < 0.0001) for both Donors 1 and 8 (Figure 4A,B). The acetate levels were similar between BMOS and the synbiotic treatments for both Donors 1 and 8. A similar finding was obtained with lactate levels in Donor 1. However, synbiotic treatments significantly stimulated the production of lactate in Donor 8 (compared to BMOS alone, Figure 4B) leading to a significant pH decrease (Figure 3). This demonstrates the positive effect of synbiotics on microbiota activity in disturbed microbiomes. On the contrary, all treatments had minor effects on ammonium levels for both donors (data not shown).

### 3.5. Preculturing B. lactis with BMOS Primed the Strain to Respond Quicker in Synbiotic Mixes

**Figure 5:**
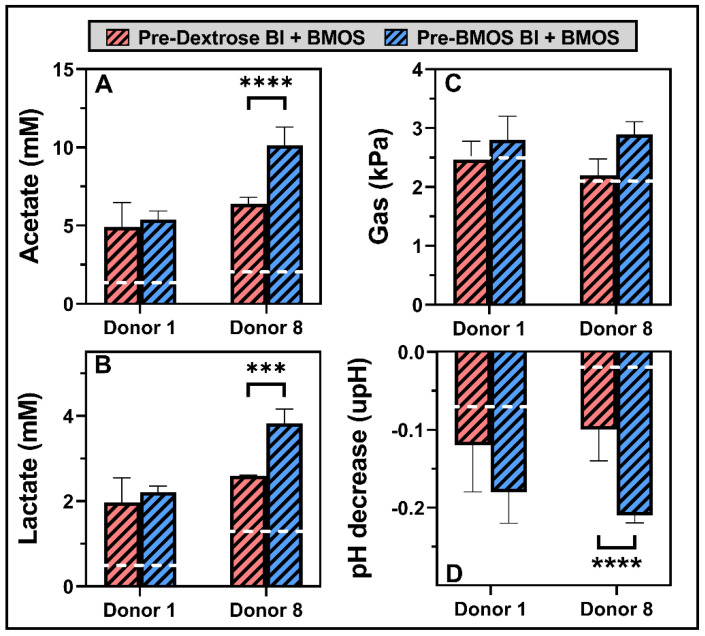
Mean (± standard deviation) microbial metabolic activity in terms of acetate production (mM) (**A**), lactate production (mM) (**B**), gas production (kPa) (**C**) and pH decrease (upH) (**D**) upon a 6 h fermentation of 5 g/L BMOS containing 1.5 × 10^7^ CFU/mL of *B. lactis* previously grown on either Dextrose (Pre-Dextrose Bl + BMOS) or BMOS (Pre-BMOS Bl + BMOS), by the infant fecal microbiota of Donors 1 and 8 (*n* = 3). For each donor, a white dotted line is indicating the average effect of the three other treatments (i.e., BMOS, Pre-Dextrose Bl and Pre-BMOS Bl); data can be found in Figure A2. Significant differences between treatments within each donor, as tested with a one-way ANOVA with Tukey’s HSD post hoc test, are indicated with asterisks (*** *p* < 10^−3^; **** *p* < 10^−4^).

**Figure A2:**
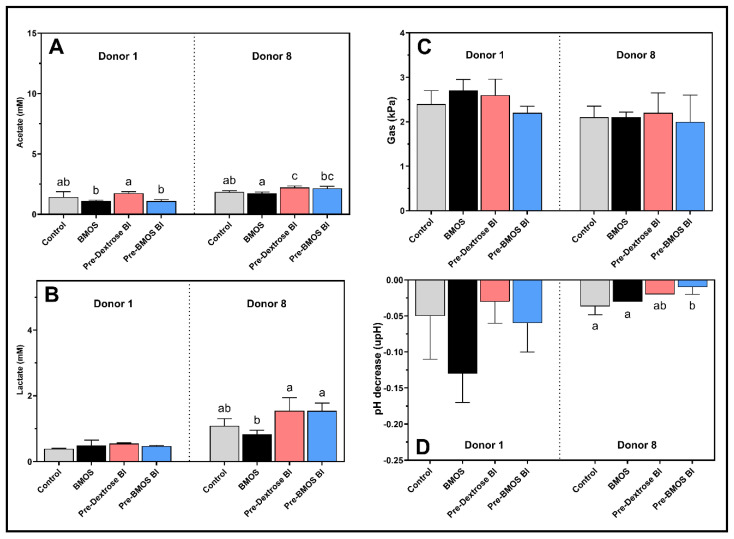
Mean (± standard deviation) microbial metabolic activity in terms of acetate production (mM) (**A**), lactate production (mM) (**B**), gas production (kPa) (**C**), and pH decrease (upH) (**D**) upon 6 h of fermentation by the infant fecal microbiota of Donors 1 and 8 after supplementation of 3 different formulations versus a blank control (Control, *n* = 3). Formulations contained either 5 g/L BMOS or 1.5 × 10^7^ CFU/mL of *B. lactis* previously grown on either Dextrose (Pre-Dextrose Bl, *n* = 3) or BMOS (Pre-BMOS Bl, *n* = 3). Significant differences between the different formulations within each donor are indicated with different letters (a, b, c) as tested with a one-way ANOVA with Tukey’s HSD post hoc test (*p* < 0.05). In contrast, when at least one letter is shared between two treatments, there was no significant difference between these groups.

All data described so far were collected after 48 h of incubation. Growing *B. lactis* on BMOS (as opposed to dextrose) did not bring any marked beneficial effect on microbiota composition (incl. *B. lactis* engraftment) and activity at 48 h. After 6 h of incubation, only minor changes occurred on pH, gas production, lactate and acetate levels in the presence of BMOS and *B. lactis* alone compared to Controls (Figure A2). However, the synbiotic formulations significantly increased the overall microbial activity of both donors during the first 6 h of incubation. Strikingly, *B. lactis* grown on BMOS and combined with BMOS (Pre-BMOS Bl + BMOS) had a significantly higher effect on acetate and lactate production (and hence the pH decrease) compared to the dextrose-grown *B. lactis* (Pre-Dextrose Bl + BMOS), in Donor 8 only (Figure 5). Therefore, *B. lactis* grown on BMOS modulated faster the microbiota activity, when combined with BMOS (synbiotic), in environments originally poor in bifidobacteria, like Donor 8.

## 4. Discussion

In the present study, using short-term colonic incubations, BMOS was shown to consistently modulate microbial activity and composition across 10 three-month old infant donors. More specifically, BMOS stimulated bifidobacterial growth, an observation already reported by Meli et al. [28] in an infant clinical trial. In addition, BMOS administration decreased pH while increased lactate, SCFA—mostly acetate—and gas production. However, cross-feeding was only sparsely observed, with light production of butyrate and propionate, as expected for preweaning infants [69]. Given the fact that a low pH has been correlated with the inhibition of pathogenic bacteria [70,71,72], and SCFA to be beneficial for the intestinal health [73,74,75,76], this study confirmed BMOS as potent prebiotic, in alignment with other studies [31]. Despite the consistent prebiotic effects of BMOS, interindividual variability was observed among the 10 donors. For an in-depth study focusing on the potential of combining BMOS with *Bifidobacterium animalis* ssp. *lactis* CNCM I-3446, Donors 1 and 8 were selected since they had respectively strong and tempered metabolic responses to BMOS treatment (e.g., pH decrease, acetate production), with Donor 8 also having amongst the lowest initial bifidobacterial count. Therefore, the microbiota of Donors 1 and 8 were different enough to reflect the variability of infant microbiota.

During the in-depth study with Donors 1 and 8, the bifidogenic effect of BMOS was confirmed using 16S-targeted Illumina sequencing. Interestingly, only a few OTUs were increased by BMOS in both donors, i.e., OTUs related to *Bifidobacterium breve*, *Lactobacillus fermentum*, *Bifidobacterium longum* and *Escherichia coli*. By contrast, OTUs related to *Bifidobacterium bifidum* and *Bifidobacterium adolescentis* were not modulated by BMOS alone. Several *Lactobacillus* and *Bifidobacterium* species have been reported to metabolize GOS—the major component of BMOS—in a strain-dependent manner [77]. Specific operons have been reported to be involved in GOS catabolism [78]. Those operons are either not constitutive, repressed or absent in some bacteria, explaining why some strains took over in the presence of BMOS in our closed system. BMOS favored the colonization of *B. breve*, *L. fermentum* and *B. longum*, which have been identified as beneficial microbes for infant health [79,80,81]. BMOS alone also favored the presence of *E. coli*. Similar findings were reported by Jakobsen and colleagues in vitro using pure GOS [82]. However, *E. coli* was suppressed in the presence of synbiotics (BMOS and *B. lactis*). Similar findings were reported in clinical studies using infant formulas enriched in BMOS and *B. lactis* [35,36,83].

Further, *B. lactis* successfully engrafted the microbiota in both donors, regardless of the initial level of bifidobacteria present. *B. lactis* is often considered as a nonresident species of the gut [39] and is only sparsely detected in infant feces [40,41]. This was confirmed in the two selected infants where no OTU belonging to *B. animalis* was detected, unless the formulations containing *B. animalis* ssp. *lactis* were given.

The synbiotic formulations significantly increased the early lactate and acetate production, observed within the first 6 h of incubation. The effect of *B. lactis* with BMOS on microbiota activity has also been reported in clinical trials in infants [35,36,83]. The synergy between BMOS and *B. lactis* CNCM I-3446 is likely explained by the fact that this strain was specifically selected for its ability to readily ferment BMOS (unpublished data). However, preculturing *B. lactis* with BMOS induced even further lactate and acetate production within the first 6 h of incubation in Donor 8. The pronounced effects of BMOS-grown *B. lactis* could be explained by an upregulation of the enzymatic machinery involved in BMOS metabolism. *B. lactis* is therefore primed to respond and grow quickly in the presence of BMOS. The effect of BMOS-grown *B. lactis* combined with BMOS on microbiota activity was significantly higher in Donor 8 than in Donor 1. Donor 8 was characterized by very low levels of bifidobacteria (in inoculum and Control). The preculturing of *B. lactis* with BMOS represents a very promising approach to strengthen the effect of synbiotics in the case of disturbed microbiota, characterized by low levels of bifidobacteria. Moreover, the speed of metabolite production by precultured *B. lactis* could be particularly important in infants where whole gastrointestinal transit times are very short and may vary between 8.5 and 10 h until 24 months of age [84]. However, more recent studies are needed [68]. Besides a fast growth speed, adhesion to intestinal surfaces is an alternative strategy to thrive in such high flow-through environments. It would be interesting to further unravel this for the specific strain CNCM I-3446, given the few studies that have demonstrated a high adhesive property of *B. lactis* strains to epithelial cells in vitro [85,86], while no such studies have been performed in vivo to our knowledge. Altogether, preculturing *B. lactis* potentially alleviates limitations in bringing health benefits due to competition, short infant bowel transit or limited cell adhesion.

## 5. Conclusions

Although this in vitro study involved only a limited cohort, it confirmed previously reported in vivo findings while providing additional in-depth insights. Inter- and intraindividual changes in microbial activity and composition between infant donors and the tested formulations were observed, which confirm the need for standardized in vitro model studies in order to better appreciate the potential differences. Administration of the probiotic alone had less potent effects than when combined with the prebiotic. This confirms the emerging interest for synbiotic formulations compared to prebiotics or probiotics alone for more predictable and guaranteed microbial modulations and potential resulting health benefits. Moreover, although still at its dawn, this study proved the benefits of preconditioning *B. lactis* with BMOS especially for short-term effects in disturbed microbiome, since it presumably increased its capacity to readily ferment BMOS. Yet, further studies would be needed to better decipher its health impact, especially on a dynamic microbial gut environment using, e.g., the semidynamic Simulator of the Human Intestinal Microbial Ecosystem (SHIME^®^) which enables the evaluation of repeated administrations of different treatments during long-term studies during which the short colonic transit time of infants can be accurately mimicked. Finally, this study confirms the large potential of applying synbiotics in infant formula, especially *B. animalis* ssp. *lactis* CNCM I-3446 with BMOS, as an effective nutritional strategy to positively modulate the development of the infant gut microbiota.

## Figures and Tables

**Table 1:** Relative average abundance (%) of different microbial Operational Taxonomic Units (OTU) upon a 48 h fermentation of 5 g/L BMOS containing 1.5 × 10^7^ CFU/mL of *B. lactis* previously grown on either Dextrose (Pre-Dextrose Bl + BMOS, *n* = 3) or BMOS (Pre-BMOS Bl + BMOS, *n* = 3), by an infant fecal microbiota of Donor 1, versus their respective controls (Control, Pre-Dextrose Bl and Pre-BMOS Bl, *n* = 3).

Phylum	Families	Species to Which OTU Is Related	Control	BMOS	Pre-Dextrose Bl	Pre-BMOS Bl	Pre-Dextrose Bl + BMOS	Pre-BMOS Bl + BMOS
Actinobacteria	*Bifidobacteriaceae*	*Bifidobacterium animalis*	0.00a	0.00a	1.00a	3.12a	21.94b	19.09b
		*Bifidobacterium bifidum*	6.07ad	8.13a	3.40b	4.07bd	6.94a	6.65a
		*Bifidobacterium breve*	2.86a	74.20b	1.75a	1.55a	55.29c	60.41d
Firmicutes	*Enterococcaceae*	*Enterococcus faecium*	17.58a	5.75bc	13.27ab	8.57abc	1.33c	0.83c
	*Clostridiaceae*	*Peptoniphilus sp.*	0.64a	0.00a	35.24b	24.55b	0.00a	0.031a
	*Lactobacillaceae*	*Lactobacillus fermentum*	0.47a	6.63b	0.32a	0.35a	9.91b	9.03b
	*Veillonellaceae*	*Veillonella atypica/dispar*	39.75a	0.00b	34.30a	25.54a	0.00b	0.04b
		*Veillonella parvula/dispar*	18.84a	0.00b	5.18c	22.25a	0.00b	0.01b

Significant differences between the different formulations are indicated with different letters (a, b, c, d), as tested with a one-way ANOVA with Tukey’s HSD post hoc test (*p* < 0.05). In contrast, when at least one letter is shared between two treatments, there was no significant difference between these groups.

**Table 2:** Relative average abundance (%) of different microbial Operational Taxonomic Units (OTU) upon a 48 h fermentation of 5 g/L BMOS containing 1.5 × 10^7^ CFU/mL of *B. lactis* previously grown on either Dextrose (Pre-Dextrose Bl + BMOS, *n* = 3) or BMOS (Pre-BMOS Bl + BMOS, *n* = 3), by an infant fecal microbiota of Donor 8, versus their respective controls (Control, Pre-Dextrose Bl and Pre-BMOS Bl, *n* = 3).

Phylum	Families	Species to Which OTU Is Related	Control	BMOS	Pre-Dextrose Bl	Pre-BMOS Bl	Pre-Dextrose Bl + BMOS	Pre-BMOS Bl + BMOS
Actinobacteria	*Bifidobacteriaceae*	*Bifidobacterium adolescentis*	0.47a	1.86b	0.17a	0.32a	0.05a	0.04a
		*Bifidobacterium animalis*	0.00a	0.00a	8.35b	3.90ab	28.57c	26.20c
		*Bifidobacterium longum*	0.24a	10.93b	0.13a	0.22a	4.30c	4.26c
	*Coriobacteriaceae*	*Collinsella aerofaciens*	0.14a	0.00b	0.07ab	0.08ab	0.00b	0.00b
Bacteroidetes	*Bacteroidaceae*	*Bacteroides ovatus*	13.63a	0.00b	1.19b	5.45ab	0.00b	0.00b
Firmicutes	*Clostridiaceae*	Member of *Clostridium* XIVa	1.53	0.00	3.50	2.96	0.00	0.00
	*Enterococcaceae*	*Enterococcus faecalis*	16.57ac	19.65ab	8.18c	8.36c	20.56ab	25.00b
	*Veillonellaceae*	*Veillonella dispar*	9.37a	0.00b	4.49ab	1.09b	0.00b	0.00b
Proteobacteria	*Enterobacteriaceae*	*Escherichia coli*	49.28a	66.82b	71.21bc	75.70c	45.97a	43.79a
		*Klebsiella oxytoca/michiganensis*	4.54a	0.30b	0.60b	0.73b	0.02b	0.02b

Significant differences between the different formulations are indicated with different letters (a, b, c, d), as tested with a one-way ANOVA with Tukey’s HSD post hoc test (*p* < 0.05). In contrast, when at least one letter is shared between two treatments, there was no significant difference between these groups.
